# Solid to cystic: A case report of imaging findings of atypical lung metastases

**DOI:** 10.4102/sajr.v23i1.1663

**Published:** 2019-01-28

**Authors:** Tanusha Sewchuran

**Affiliations:** 1 Department of Radiology, College of Health Sciences, Nelson R. Mandela School of Medicine, University of KwaZulu-Natal, South Africa

## Abstract

The imaging spectrum of pulmonary metastases varies greatly, with solid and partly cavitating nodules being the most common. When imaging the oncology patient, specifically follow-up imaging post-treatment, the radiological aim is to assess for disease regression and thus treatment response, usually with resolution of these nodules. We report an interesting case series of a patient with primary endometrial carcinoma presenting with pulmonary metastases. This imaging series eloquently depicts the temporal evolution of the metastatic solid pulmonary nodules to cavitating nodules and finally to thin-walled cysts. Baseline imaging in this scenario is vital to exclude pre-existing cystic lung disease. The progression of solid pulmonary metastases to simple cysts is an uncommon therapy-related consequence, but an important entity to recognise, not only as an indicator of good treatment response, but also to evaluate for potential life-threatening complications such as spontaneous pneumothoraces.

## Introduction

The radiological imaging spectrum of pulmonary metastases is vast and may range from solid nodules to lesions mimicking pulmonary cysts. The histology of the primary tumour is often helpful, particularly in the setting of squamous cell carcinomas, where primary and secondary pulmonary lesions often appear to be cavitating.

## Case report

We report an interesting case series of patient KM, a 64-year-old female who had presented with a 4-month history of post-menopausal vaginal bleeding. Her co-morbidities included senescent hypertension and cardiac disease, stable on double agent anti-hypertensives. She was also a social smoker. An initial pipelle biopsy sent for microscopy revealed moderately differentiated carcinoma in the endometrial tissue which stained positive for vimentin and oestrogen receptors, but negative for carcinoembryonic antigen (CEA). This was consistent with endometrial adenocarcinoma. She subsequently presented to radiology for staging computed tomography (CT) scan post-surgery (bilateral salpingo-oophorectomy and total abdominal hysterectomy). Her post-operative recovery was volatile with ureteric reimplantation required after iatrogenic injury, several urinary tract infections and renal complications. Patient KM’s initial CT abdomen/pelvis, with imaging through the lung bases, revealed multiple bilateral solid pulmonary nodules of moderate size, in keeping with Stage 4 disease. The distribution was random with no associated calcifications, septal thickening or pleural effusions. She received single agent palliative chemotherapy with Paclitaxel 280 mg, a taxane-based chemotherapy agent targeted for the treatment of endometrial adenocarcinomas. The usually used combination treatment of a cisplatin was not utilised owing to her ongoing renal dysfunction.

**FIGURE 1:**
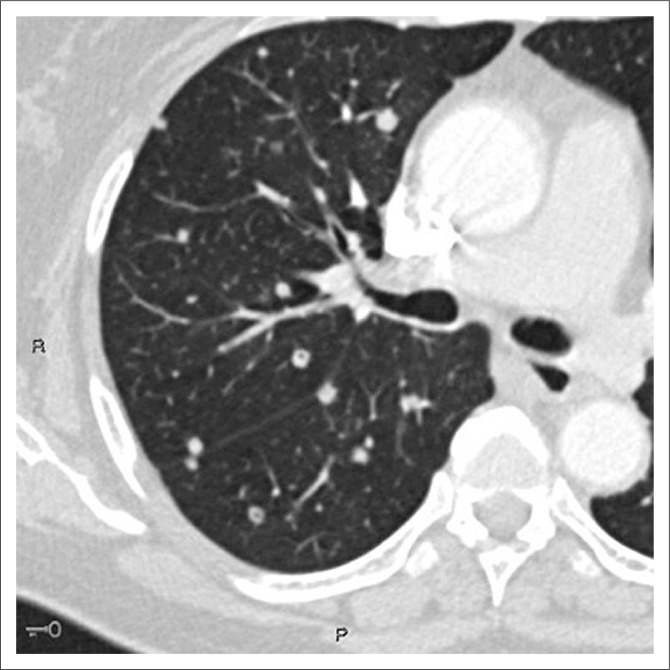
Computed tomography axial image through the right lung base demonstrates early central cavitation of the pulmonary nodules.

**FIGURE 2:**
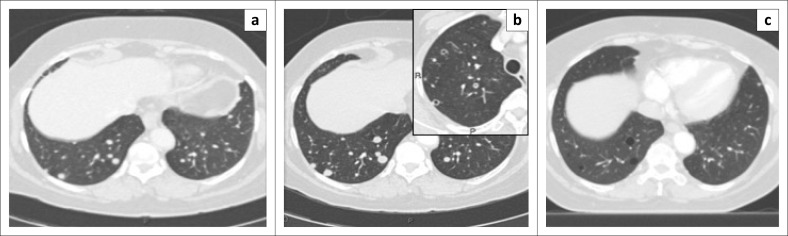
Consecutive computed tomography axial image series through the lung bases eloquently depicts the temporal evolution of: (a) solid pulmonary nodules to (c) thin-walled cysts. The magnified insert clearly demonstrates the central cavitation (b).

**FIGURE 3:**
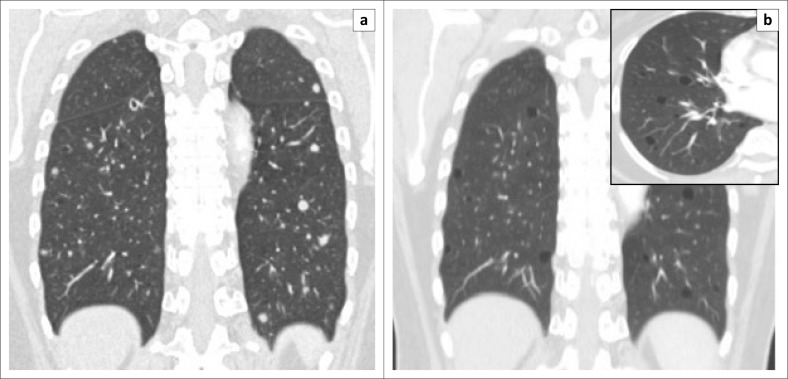
Coronal multiplanar reformatted images depicts the progression of solid pulmonary nodules to: (a) thick-walled cavitating nodules to (b) thin-walled pulmonary cysts over time. The uncomplicated thin-walled cysts are better appreciated in the magnified insert (b).

On dedicated chest imaging, approximately a month after initial staging and initiation of treatment, several of the previously noted solid pulmonary nodules now demonstrated discrete central lucencies, in keeping with cavitation. Figure 1 eloquently demonstrates this central cavitation. Upon completion of the six cycles of chemotherapy, the final staging scan performed approximately 3 months later exhibited good treatment response with disease regression. All the afore-documented solid and early cavitating pulmonary nodules had now resolved to thin-walled cysts with no associated soft tissue component, calcification or ground glass attenuation. No pneumothorax was present. Figure 2 depicts the temporal evolution of several focal right lower lobe solid pulmonary nodules to thin-walled cysts on axial CT images. In Figure 3, we can also clearly appreciate this cavitating trend on coronal multiplanar reformatted images.

## Discussion

Prior to a decade ago, primary treatment for patients with uterine cancer was radiotherapy based.^1^ Chemotherapy was reserved for patients with recurrent or disseminated disease, especially in the setting of failed hormone therapy.^1^ The use of taxane-based chemotherapy agents has been documented as being highly effective in the treatment of adenocarcinomas.^2^ Together with platinum agents, such as cisplatin, it is often the first-line chemotherapy agent used. In the documented literature, a patient known with primary adenocarcinoma of the lung, who was treated with Docetaxel, demonstrated secondary cavitatory change of the pulmonary metastases with bilateral spontaneous pneumothoraces.^2^ Docetaxel is also a member of the taxane family of chemotherapy agents. Paclitaxel, the drug of choice in patient KM’s case, demonstrated good treatment responses in advanced/recurrent disease.^1^

The lung is the most common location for metastatic lesions from a non-pulmonary primary malignancy.^3^ Pulmonary metastases via haematogenous or lymphatic spread is usual, with cavitating or cystic appearing pulmonary metastases considered a subgroup.^4^ This makes the distinction from incidental non-malignant lung pathology much harder.^5^ The clinical circumstances and course of disease, that is, an acute onset versus protracted illness, are important considerations when evaluating cystic and cavitating lung lesions.^3^

According to the Fleischner glossary of terms, a cyst is defined as a ‘round circumscribed space surrounded by an epithelial wall of variable thickness’.^6^ On imaging, it appears as a rounded lucent lesion and is usually thin-walled.^6^ In comparison, a cavity is a lucent lesion within an area of consolidation or within a nodule.^6^ A nodule is classified as a rounded opacity measuring up to 3 cm, and may appear poorly or well-defined.^6^

Cavitation of solid pulmonary nodules may be spontaneous or therapy-induced, from either chemotherapy or radiotherapy.^7^ Upper lobe and centrally located lesions are more likely to cavitate.^7^ Whilst squamous cell carcinomas are renowned for cavitating pulmonary lesions, other possible primaries include sarcomas, and gastrointestinal, bladder or pancreatic malignancies.^4,5^ It has been well-documented that certain chemotherapy agents are known to induce cavitation.^1–3^ Several postulated mechanisms included tumour necrosis or insinuation of air with a check-valve mechanism into the tumour.^1–3^ Spontaneous pneumothoraces have also been reported in previous literature, citing increased intrathoracic pressures following emetic sequelae of chemotherapy or possible bronchopleural fistulae formation from tumour lysis/necrosis, as aetiologies.^2^ Rupture of sub-pleural bullae or emphysematous bullae have also been postulated.^8^ Such patients were subsequently treated with closed chest tube insertion and chemical pleurodesis.^2^

## Conclusion

The evolution of solid pulmonary metastases to cavitating and then cystic lesions, as a consequence of therapy, is a fairly rare entity albeit an important one to consider. The possibility of spontaneous pneumothorax is an uncommon but significant complication, which requires interval clinical and imaging evaluation to preclude preventable patient morbidity and mortality.
